# Patients’ Experience of Medication Brand Changes during Hormone Therapy for Breast Cancer—An Interpretative Phenomenological Analysis

**DOI:** 10.3390/healthcare10122558

**Published:** 2022-12-16

**Authors:** Yolanda Eraso, Zoe Moon, Ieva Steinberga

**Affiliations:** 1 Centre for Primary Health and Social Care, School of Social Sciences and Professions, London Metropolitan University, 166-220 Holloway Road, London N7 8DB, UK; 2 School of Pharmacy, Faculty of Life Sciences, University College London, Gower Street, London WC1E 6BT, UK

**Keywords:** breast cancer, hormone therapy, medication adherence and persistence, generic drugs, side effects, interpretative phenomenological analysis

## Abstract

Medication adherence to hormone therapy (HT) in breast cancer survivors is often suboptimal and is affected by a range of factors. Patients are usually prescribed different generic formulations of HT drugs and their impact on side effects and on adherence and persistence is poorly understood. This study aimed to explore women’s lived experience of HT medication brand changes (generic substitution) and its impact on side effects, quality of life and medication-taking behaviors, as well as on adherence and persistence. Nine female breast cancer survivors who had previous experience of HT medication brand changes participated in the study. Individual, online, semi-structured interviews were conducted and analyzed using interpretative phenomenological analysis. The findings identified three superordinate themes and nine subordinate themes that influenced the lived experience of medication brand changes for these patients: (i) experiencing brand changes, (ii) responsiveness of health care providers and (iii) future expectations. Women reported negative physical and emotional experiences of brand changes, which is often compounded by healthcare professionals’ lack of information and reassurances, disbelief in the worsening of side effects and inconsistent advice regarding generics. These have implications for women’s self-efficacy for medication-taking behaviors, ability to manage side effects and HT adherence and persistence.

## 1. Introduction

Breast cancer is the most common cancer in the UK, with an estimated 55,500 new cases being diagnosed in women every year [1]. Around 75–80% of breast cancers are estrogen receptor-positive, and, after the initial treatment of the tumor, adjuvant hormone therapy (HT) drugs are prescribed for five to ten years to reduce the risk of recurrence. HT drugs include tamoxifen and aromatase inhibitors (AIs) (anastrozole, letrozole, exemestane), all of which are known to cause burdensome side effects affecting patients’ quality of life.

Non-adherence to HT (not taking medication as prescribed) and non-persistence (early discontinuation of treatment) is around 50% by the fifth year of treatment [2,3,4]. Systematic reviews have identified self-efficacy in medication-taking and positive beliefs about HT (necessity/concerns framework [5] as potential predictors and modifiable factors for adherence and persistence with HT [6,7].

Our previous mixed-methods study about women’s decisions on extending HT to 10 year, analyzed 130 women’s discussions on the UK ‘Breast Cancer Now’ online forum and revealed self-efficacy in managing side effects as a statistically significant factor influencing treatment continuation. Remarkably, changing generic brands of HT drugs was one of the strategies that allowed some women to manage side effects, regain quality of life and continue with treatment [8]. In the UK, the use of generic HT drugs and their impact on patients’ experiences with treatment and on adherence and persistence has been scarcely studied. The National Institute for Health and Care Excellence (NICE) guidance on prescribing states that non-proprietary (generic) titles should be used and that only those medicines with proven differences in clinical effects should be prescribed using the brand name or the manufacturer’s name [9]. HT drugs are not exempted, and therefore it is common practice for pharmacists to dispense a different generic to patients each time they collect their prescriptions. Currently, there are 10 generics for tamoxifen (20 mg, most commonly prescribed dose), 10 generics for letrozole (2.5 mg) and 17 for anastrozole (1 mg) [10].

The literature on patients’ perceptions and acceptability of generic drugs have largely been conducted for anti-epileptic drugs, immunosuppressants and antidepressants and have shown variations in medication-tacking behaviors and in adherence and persistence. Patients’ negative perceptions toward generics have ranged from nocebo effect and emotional drivers; doubts about quality and effectiveness (for being less expensive); and attribution of new side effects to apprehension toward the color, taste and shape of the new compounds [11,12,13,14]. Studies have also found significant risk of discontinuation in first-time users of generics and in those changing medication brands at first refill [15,16]. Furthermore, research on patients’ perspectives on generics has consistently reported that improving knowledge and information about generics by physicians or pharmacists can increase consumers’ confidence and acceptability [12,13,17].

In contrast to those conditions, patients’ experiences with generics in HT for breast cancer and its role in adherence is an under-researched area. A few articles in the US have focused on the introduction of generic AIs and found an increase in adherence due to a reduction in co-payments [18,19,20,21]. A study by Quin et al. [22], however, argued that non-initiation of treatment remained high after the introduction of AIs generics, contending that lower prices did not fully explain access to AIs, and suggested instead that other factors such as doctors’ prescribing behaviors, patients’ perceptions of generics or pharmacies making drug substitutions could better explain treatment initiation and drug preferences. In the UK, only two studies have investigated the impact of tamoxifen generics in HT. The first one, surveyed hospital patients in Hampshire where 63 patients were aware of changing brands, of which 77% reported suffering severe side effects, and a small but significant (11%; *p* = 0.02) number of patients attributed a difference in severe menopausal symptoms to the brand changes [23]. The study concluded that women who changed generic brands were more likely to consider discontinuing compared to those who stayed with the original generic drug [23]. The second study was developed following on anecdotal information of some women on generic formulations of tamoxifen who reported symptoms of arthralgia, a side-effect that was absent in the brand-name Nolvadex^®^ [24]. A prospective design was conducted in an NHS Foundation Trust in England recruiting 918 women who initiated HT on generic tamoxifen (four brands available at the time) and reviewed at 6 months for musculoskeletal symptoms. Patients who reported arthralgia (13.2%; 121) consented to switch to Nolvadex^®^ for 6 months, at which point 94.2% (114) patients reported an end to their joint pain symptoms (*p* < 0.05). Interestingly, 116 of these women were put back on the generic version for six months and all experienced arthralgia. The study concluded that a small percentage of women (13.2%) suffered from joint pain while on generics and that this perceived difference from branded tamoxifen was probably excipient-related, rather than being driven by patient perceptions or the nocebo effect [24]. Both UK studies reported a small but significant percentage (11–13%) of women who report symptom changes associated with different brands of HT. Anecdotally, many women report that they can only tolerate certain brands on HT, but very little is known about women’s experiences of this and how they cope with HT brand changes.

In this study, we aim to produce empirical data most directly relevant to support breast cancer patients who noticed worsening of side effects after HT changes in medication brands. We addressed two broad research questions: how do patients experience medication brand changes for HT? and how do medication brand changes affect their medication-taking behavior, adherence and persistence with HT? Our findings identify the impact of medication brand changes on women’s self-efficacy for medication-taking behaviors, on their ability to manage side effects and on HT adherence and persistence.

The rest of the paper is structured as follows. In the next section, we describe the study design and methodology. Then, we present the superordinate and subordinate themes alongside extracts from participants’ accounts. The last section offers a discussion and conclusions with information on the development of supportive interventions.

## 2. Materials and Methods

### 2.1. Design

A semi-structured interview was used with each participant, and interpretative phenomenological analysis (IPA) informed the design of the study [25]. IPA is an idiographic, experiential approach of individuals’ accounts of their lived experience that has been extensively used in health research. We felt that IPA was the preferred qualitative approach primarily because medication brand changes for HT has not been explored yet and that an experiential approach would help us capture women’s experiences in a double hermeneutic way, i.e., the voice of the participant’s sense-making and the researchers’ interpretation by making sense of participants’ experiences about this phenomenon. This approach has the ability to bring to light relevant issues around medication brand changes to healthcare professionals and contribute preliminary in-depth qualitative work to inform future interventions based on a person-centered perspective (i.e., identification of patients attitudes, needs, challenges and preferences to address) [26]. Therefore, it was deemed advantageous in relation to other qualitative methodologies.

The consolidated criteria for reporting qualitative research (COREQ) was used as reporting guidelines of the study [27], (see File S1).

### 2.2. Participants and Procedures

Participants were recruited via the Breast Cancer Now online forum in the UK [28], after following the charity’s procedure for researchers to recruit participants. Our recruitment strategy encompassed two topics in women’s experiences of HT: women with experience of medication brand changes and/or experience in the use of prognostic tools to help them decide about treatment continuation. There were 12 participants who responded to the call, but after contact, only 10 agreed to schedule an interview. For this study, nine women were purposely selected as a homogeneous sample of women with previous experience of medication brand changes. This sample size concurs with other IPA health studies [29,30,31], wherein the emphasis was to provide a detailed interpretative account of the perceptions and understandings of the participants included.

Interested participants were provided with an online participant information sheet and were requested an email for contact with the research team and to state their preference for phone or videoconference interview. Individual interviews were conducted online via Microsoft Teams and Zoom (*n* = 7) and over the phone (*n* = 2) on 9 July–19 September 2021 and lasted a mean of 37 min (range 24–63 min). Participants were all living and treated in different parts of England and had a mean age of 57 years. Table 1 presents more details of participants and their HT status.

The semi-structured interview schedule was designed by the authors as open-ended questions with prompts and was informed by our previous research on the Breast Cancer Now online forum and the broader literature on patients’ attitudes when switching generic drugs. Key questions included: Have you ever changed the brand of your medication? Was this recommended by your healthcare professional, or did you ask your healthcare professional to change it? How was your experience with the new brand? Do you recall any problems in accessing the brand that your health professional recommended or that you requested? How did changing your medication affect your decision on whether to continue taking your HT drugs? In your experience, how could changing medication brands be improved?

Interviews were audio-recorded, transcribed verbatim and pseudonymized for each participant. The interviewer (IS) was female, with a background in reproductive sexual health and experience in qualitative interviews. The other authors are qualitative research specialists with expertise in breast cancer adherence to HT with a background in psychology and health studies. None of the researchers had a previous relationship with the participants.

The study received ethical approval (ID 4050119) from the School of Social Sciences and Professions Research Ethics committee at London Metropolitan University. Informed consent was obtained from all participants.

### 2.3. Analysis

Data were analyzed following the IPA guidelines. Each interview recording was transcribed verbatim by IS. YE and IS took the lead on the analysis, by independently reading and rereading accounts and in some instances returning to recordings before line-by-line coding and making notes of emerging topics. After each interview was annotated with themes and subthemes, the analytical work continued with the next interview. We compared and discussed our themes and subthemes alongside our personal reflections of each interview. After agreeing on themes to be included/excluded, YE prepared a first draft of the analysis. The latter was shared with ZM, who had read all transcripts and took on an audit role, proposing further renaming of subordinate themes and a rearrangement of chosen quotes. Discussions continued until an analytical consensus was reached, ensuring that the breadth of transcript convergence and divergence had been captured. The selection of themes and sub-themes followed Nizza, Farr and Smith’s [32] recommendations for high-quality IPA. Our initial deep reading of the data was facilitated by making the double hermeneutic (interpretative engagement) more complex and integral amongst the research team, an IPA analytical process we can describe as closer to ‘multiple hermeneutics’ [33].

## 3. Results

The analysis elicited three superordinate themes and nine subordinate themes, presented in Table 2. The name of drugs has been pseudonymized with 2 capital letters followed by *.

### 3.1. Experiencing Brand Changes

All women were aware of having the brand of their HT changed, and seven out of nine had a negative experience after the brand changed. Participants’ experiences are organized into three subthemes: feeling the change in brands, losing control over treatment and motivation to continue.

#### 3.1.1. Feeling the Change in Brands—Impact on Wellbeing

Many women felt the physical and emotional impact of changing brands. One woman referred to the physical changes experienced when switching from brand name TAM to generic formulations:

*‘Well, I had side effects with tamoxifen right from the beginning and it was worse as I changed to generics. And it was complete lethargy, absolute complete lethargy’* (Mary).

Another participant assertively commented:

*‘Oh yes, [smiling] yes, yes. I discovered the different brands give you quite different reactions’* (Diane).

One woman was doubtful about attributing the new side effects to the generic switching:

*‘No, not that I have noticed. I think…well, you see it has not really been long enough because I started in January. And the new packet only came about a month ago. So maybe I’ve only had 4–5 months with one packet and another month with another. So maybe haven’t had long enough to notice’* (Rose).

Another thought that changes in brands did not make any difference to how poorly she felt while taking HT:

*‘For me, I wasn’t aware of any difference in the brands. May have been or it may not, I couldn’t say, but for me just the antiestrogen effect is so awful that I am not sure that the brands differences would make much of a difference if you know what I mean?’* (Sue)

Women experiencing worse side effects became very aware of which specific brand was not acceptable or tolerable to them and retained the manufacturer’s name for future interactions with healthcare professionals and pharmacists.

*‘That month was horrible, with the tamoxifen, I had a particular month but with much worse [side effects] than the one before, and then it was fine again and I felt Uhhh… so let’s see then so, you know, I did a little experiment and I did keep a note and kept my boxes so I knew which ones I got, that’s what my body made me decide that I just didn’t want that particular brand’* (Helen).

The expression ‘my body made me decide’ resonates with the need for women to justify their physical experience of changing brands rather than a psychological, nocebo-type effect that often healthcare professionals believe in when patients request or reject a particular brand.

Other women also clearly identified the brands that were right or wrong for them, discriminating between brands by degrees of severity experienced:

‘So, the first change [pause] I think I did notice it, but it wasn’t too extreme, but it was when… it was this one where it was very extreme, it’s TG*’ (Kate).

‘I’m happy with RG* think it is, that’s fine. I think it’s WG* gives me really bad knee pain, so I avoid WG*’ (Liz).

Women also referred to the significance of getting used to and being able to cope with the side effects of one drug, the impact of new side effects after changing medication and the emotional consequences that some experienced because of this process.

‘I just had so many more side effects [new generic] and also swapping…. swapping brands has an effect on me emotionally… sort of how you feel, and I mean, so I was just expecting the WG* one, and these are the side effects and you get used to it, then again a new brand […].

*Because… just because when they change brands, I mean there’s the side effects, it does affect your mood anyways, but for me it’s like for two weeks…the world seems very… sounds stupid, very bleak and everything just seems very hard and difficult to cope with. And since they stopped the brand on me, other than the other side effects, that’s what you are coping with and on top of… I mean I’m slightly further away from breast cancer now, but when you first get it…you know, I was going through radiotherapy and you’ve just had surgery, and it’s like… you look at things and you think ‘oh I am fine now’. It takes several months to have come to terms with things and get your head around it and to sort of have the change, you know the impact of the changing pills on top of that is actually quite a lot. And people, I guess me, before you have cancer you don’t realise the mental impacts, the long-term impacts. You know, you just think about: ‘Oh you have this operation, you have this treatment, and then fine.’ But.. well, then you know it’s really not like that’* (Anna).

We can perceive a sense of injustice from Anna as she recounted going through a difficult journey wherein patients are evolving in their cancer survivorship from active care to post hospital discharge. Changing medication at this point in their cancer experience, especially after getting used to and coping with the side effects of one brand, appears to be having an emotional toll.

#### 3.1.2. Losing and Regaining Control over Treatment

When medication was changed for the first time, women reported being initially surprised and then losing control or agency over treatment. Most of them commented on the lack of previous information regarding the dispensing of generic substitutions when starting their HT medication:

*‘It was just what I was given at the pharmacy. It wasn’t like … nobody said you will be taking a different brand, or you know, but it was just what I was given’* (Kate).

Kate feels the change as an imposition, something done without her having any knowledge or say over the drugs given. The same sense of lacking agency was expressed by Mary, who tries to explain what she later found out about the reasons for changing her medication from brand name to generics:

*‘Well, as I said, I was under the assumption, I mean I can’t remember having that conversation, they [GPs] were told they have to give generics because they were cheaper. In a way I didn’t have an option unless I started paying for it’* (Mary).

Another participant commented about her first encounter with a different brand:

‘It actually happened by accident. So, I don’t know if you’re aware that the pharmacists, if you say tamoxifen, they just give you whatever brand they have in stock. So I started off with WG* tamoxifen which is almost quite lucky because it is a brand I had the fewest side effects from. And then the next month, just because it said just tamoxifen again, they gave me TG*. It just had so many more side effects’ (Anna).

By equating changing with ‘accident’, Anna conflates the notion of unexpected and unintended harm, as she reckons with new side effects after the substitution.

Most women who experienced worse side effects with the new brand tried to get hold of the brand they knew worked better for them. Some of them became more assertive as they were motivated to find the right brand. As described by Diane, the process is sometimes lengthy and requires perseverance:

*‘I went round, and this is typical of a number of my friends, we’ve all gone round various pharmacies saying: what brands do you have, what can you get, and some chemists say, ‘we get whatever the suppliers send us, we can’t ask for something’. So there is a lot of trial and error until I found somebody [a pharmacist] that was sympathetic’* (Diane).

In the case of Anna, she urged her GP to change the prescription to her preferred brand, but the pharmacy stated it could not source that formulation anymore. She then turned to the drug manufacturer, whereby she found a way of accessing the drug for which she feels fortunate:

*‘So actually, I contacted the manufacturer to check what they [pharmacy] were telling me. So coming from the pharmaceutical industry sort of help things, so they told me that it has stopped manufacturing 20 mg, that it was not available and what was available. So that I actually said to [name of pharmacy]: ‘But the 10 mg. is available’. They said, ‘go back to the GP to get your prescription change so you get it’. So, I was very focused on what I wanted, basically [laughing]’* (Anna).

#### 3.1.3. A Motivation to Continue with Treatment

Most patients considered that having the chance to change a medication that was not suitable to them had a strong association with their continuation. Specifically, they were asked how helpful was for them the ability to change medication brands for their decision to continue with HT:

*‘Yes, I think it is very important, you know, and I do feel if someone feels that the proper branded drug is good for you, they should be allowed and not force you into generics’* (Mary).

Mary is reflecting on her experience when branded tamoxifen was discontinued and only generics were offered. She eventually discontinued tamoxifen due to side effects and was offered AIs. The extract here shows the relevance of patients’ agency over their medication in motivating them to adhere to treatment. Similarly, other women commented:

‘Oh yes, Oh yeah, I thought if I continue having to take WG* I’m gonna have a real problem about continuing, but finally I found one [brand] that is not too bad’ (Diane).

*‘I think it’s important because if you find the right one then I think you can stay on it for longer’* (Liz).

The expression finding ‘the right one’ encapsulates the feeling that the drug needs to be tolerable to the patient and not the other way around.

Meanwhile, Kate stated that she stopped taking a particular brand due to side effects, but she was then tolerating another one, so she continued. It is worth considering the impact this medication-taking behavior has not only for persistence but for adherence too, as women may skip doses and wait for their preferred brand to arrive.

‘Well, I know one that was awful, but I’m afraid…I actually saved this [package] cause I refused to take the final one. The side effects were so horrendous with this one and…it’s TG* I’m not taking it’ (Kate).

### 3.2. Responsiveness of Health Care Providers

All participants talked about experiencing a negative response when engaging with healthcare providers, while others considered themselves fortunate when reflecting on positive interactions. Their experiences were grouped into three subthemes: feeling abandoned by the health system—lack of continuity of care, encountering disbelief and feeling lucky.

#### 3.2.1. Feeling Abandoned by the Health System—Context of Lack of Continuity of Care

Many of the women who suffered worse side effects after changing generic brands reflected on the lack of access or lack of meaningful encounters with their breast cancer team after being discharged from the hospital:

*‘I’m on this, sounds really weird, it sounds really weird to me, but I’m on a five-year survival plan with the breast care unit. And I meant to have like open access to them, but the two times I have tried to get in touch with a breast care nurse, I have not heard anything back. And I just think they’re so busy’* (Kate).

The use of the word ‘weird’ in this fragment suggests her disappointment after an unmet expectation, i.e., accessing health advice for something as meaningful as a ‘survival plan’. Furthermore, Kate, similar to many of the patients, offers a resigned response when considering staff as busy or having more critical patients to assist.

For Diane, the transition to primary care was also challenging:

*‘I felt also that you know once my treatment was over, that was it, and everybody I’ve spoken to had the same feeling that suddenly it all stops and you’re left on your own to cope, and psychologically… I am quite a resilient person but that was very, very difficult’* (Diane).

The transition from acute specialized care to primary care was experienced by Diane as a sudden stop in ‘treatment’ as if, for the health system, there was no more treatment to continue with. Except that there was (HT), and she found herself alone to cope and keep going, inhabiting the cancer care continuum as a treatment/non-treatment oxymoron.

Another participant expressed:

*‘I haven’t had a great experience with them in the four years I’ve been under them, so I wasn’t overly keen to keep going. All it did was stress me out going to appointments where nothing really happened and it was only the last one where I said no, I want to do the talking at this one. […] the only time I mentioned that I didn’t like the side effects it was just in passing [...] and I said oh you know the side effects of tamoxifen and then he just said ‘Oh well they’re all, they’re all much the same’* (Helen).

The yearly appointments with the breast cancer team were seen by Helen as a top-down encounter. When she took an active role in the conversations to raise concerns about side effects, it left her disappointed and refusing to come back, despite her many current concerns about her medication.

#### 3.2.2. Encountering Disbelief

One of the responses that participants often received from healthcare providers was that generic drugs were not different from each other or in relation to the original brand. This response left women feeling that healthcare professionals often mistrusted them and that their concerns were downplayed or dismissed.

*‘Well, I started off with my GP and the GP said we don’t request particular brands because we don’t want to be associated with a particular pharmaceutical company, and I understood that, but then, then they said, I spoke to two different GPs, they said is the same formulation so you shouldn’t have any difference’* (Diane).

*‘I think if there would be more understanding, awareness that different brands does make a difference. Because I did feel like the pharmacist didn’t really believe me’* (Anna).

*‘I think you’ll find most doctors, I am not sure about nurses, but most doctors will say there is no difference in brands, I mean actual brands, names, but most doctors would say there is no difference’* (Sue).

Diane and Anna encountered disbelief from healthcare professionals when complaining about different side effects from drugs as related to a combination of lack of knowledge and lack of empathy with patients’ experiences. This combination lays bare the complexity underpinning the phenomenon as it sits at the crossroad of two assumptions: the scientific principle that states that generics and brand name are the same drug, as described by Sue, and a patient-centered approach, wherein patients’ experiences with their medication, preferences and views in self-managing the disease is not properly acknowledged.

Interestingly, some of the women also reported that until they experienced it themselves, they were also sceptical that the brands could have an impact on side effects.

*‘I was actually shocked how different the side effects are between different brands because you know if people said it to me before I was sceptical’* (Anna).

This disbelief leads to some healthcare professionals being reluctant to support women with their requests to stick to a particular brand, leading to feelings of frustration as narrated by Julie.

*‘Actually, I think, and I don’t know why this happens to a lot of people but I have thought about it myself that, you know… the pharmacy a little bit like […] they are the gate keeper to your drugs, and to be honest at one point when I went for very early on you know, you just think: aren’t we going through… haven’t we been through enough to not just give us the thing? We don’t even want to take the… you know, who wants to take letrozole? But it’s a must, but surely you can give us the one we want’* (Julie).

Julie, as seen in other extracts, needs to draw on a women-shared experience first before allowing herself too deep into hers, as a way of signalling the authentic experience (‘going through’, ‘enough’) she valued too highly to be disbelieved.

#### 3.2.3. Feeling Lucky

Women who managed to change their medication brand by involving a healthcare professional in an uncomplicated manner tended to consider the help received as being lucky and exceptional. They also extended this feeling to being able to continue with treatment.

*‘When I got really, really bad knee pain, like for a long time, I rang my doctor and I said could it be the tamoxifen? And like if it is, can we change the brand? And he got the delivered straight away. [...] I don’t know if he agreed necessarily, but he definitely went along with it’* (Liz).

Liz called her GP after attributing a new pain to a different brand; she confirmed that the pain subsided after changing the medication and considered this proof of a specific side effect brought by the medication. She is, however, unconvinced that her GP believes in the medication causing it, but she is pleased that her claim was considered.

*‘I ended up with a pharmacist, that you know, as I said, after quite a lot of trial and error, a pharmacist who is very sympathetic’* (Diane).

For Diane, finding someone ‘very sympathetic’ is highlighted as an exception after consulting with GPs, pharmacists and a difficult experience with the breast cancer team, to which she referred as an ‘uninterested’ oncologist and a ‘far too busy’ nurse. Making sense of the impact that medication change has had for her treatment continuation, she contrasts her favored situation with that of a friend who could not access a suitable brand or professional advice on potential choices. She added:

*‘I’ve got one friend in the group that I mentioned to you, who just stopped it altogether because she couldn’t find a brand that was supportive to her, so she stopped. But again, she was like me, she couldn’t get a discussion going about what the differences might be’* (Diane).

By saying about her friend, ‘she was like me’, she implicitly identifies her continuation with treatment as a matter of good fortune.

### 3.3. Future Expectations

All participants discussed their future expectations with medication-taking for HT and how support could be improved. This master theme encompassed three subordinate themes: needing information about brands, seeking specialists’ reassurances and managing prescriptions.

#### 3.3.1. Needing Information about Brands

Women indicated the importance of early and consistent information about the likelihood of receiving different brands of the same medication, and with it, the possibility of experiencing different side effects.

*‘Well, possibly cause it all comes through my GP now… And… My GP has been great. So, I think that I needed to be told that I could… It’s kind of like… I should be able to say: ‘No, this brand is really, I’m really not dealing with it well. I don’t want it again. Can I have…?’ There’s just been no dialogue about it, so it’s… I think yeah, because you know my prescription is issued by my GP… I should know that I can talk to them’* (Kate).

Kate is reflecting on her perceived right to exercise choice and have a say on the medication she is taken by making suggestions to her prescriber (GP) of which brands she thinks work better for her. She feels as if that agency has been withdrawn from her.

The timing of providing this information, according to Liz, should be at the point of hospital discharge. She refers to the value of anticipating risk and avoiding unnecessary side-effect-induced ‘panic’ during HT, alongside the provision of information about brand options.

*‘I think it would be helpful at a stage of breast care nurse, oncologist, something like that to mention to you…this might happen because nobody said it to me emm…or gave any options and you know, like if you don’t get on with this one [brand] there’s that one, or told me like to watch out for anything. Nobody said that. And I think that would have been helpful because sometimes you just panic, you know, like you think you’ve got like new symptoms and stuff. Whereas actually if you know that there might be other options, it would be better’* (Liz).

A more challenging point is raised by Julie, who was advised to always take the same brand by her oncologists. This illustrates the lack of consistent information among healthcare professionals when advising women about brands, offering them less opportunities to be in control of their medication, as narrated by Julie:

‘So when I mentioned a side effect to the oncologist at my six-monthly review, he said: “Always stick to the same brand”, then AR*brand name. Between my GP and the pharmacy…emm they told me that it’s something to do with costs and…emm availability. So, to be honest, I just started taking what they gave me […]. I would say: “Can I have the AR* brand name please?” and they would say: “Well, we’re not allowed to do that”. And to be honest, I’ve been backwards and forwards so much about trying to check that I just gave up’ (Julie).

#### 3.3.2. Seeking Specialists’ Reassurances

There was something of a paradox for participants being put on a long-term medication regime without being able to rely on specialists (oncologists or breast cancer surgeons) for further reassurances on the individual benefits of HT and the ensuing side effects after changing brands. This expertise was often sought when a request for specific brands was made to GPs and they referred women back to their breast specialist.

*‘But it is to just actually have the support and I think that they [oncologists] weren’t interested or bothered. But to expect you to stay on it [medication] but giving you no support’* (Anna).

When Anna explained in a letter to the registrar that she experienced seven or eight side effects after changing to a new generic, including thinning of hair, dizziness, mood changes and tiredness, she recalls, ‘they said to manage the joint pain is the only medical side effect’.

The lack of specialists’ support about prescribed medications is seen by Helen as having more serious consequences for patients’ decision making about treatment:

*‘Believe me, you just don’t want to have to go and row with the pharmacy and they don’t want to row with you, their life is miserable as well at the moment. It’s the feeling that you don’t have anyone to talk to, I think that’s the issue. And at the moment people can’t make an appointment anyway, they say “there is none to talk to”. So at the moment people will just be making their own decisions based on anything and nothing which is really worrying’* (Helen).

She is rightly concerned about the COVID pandemic as complicating access to services even further. Yet her dual position as patient and pharmacist allows her to frame the discussion about brands as a ‘row’ that neither patients nor pharmacists want, portraying them both as unintended sufferers of the lack of reassurance from the breast cancer team.

Rose also asserted how relevant information is about risks and benefits for patients making an informed choice on their medication. In her case, she linked the existence of different brands and the lack of information about them with the broader lack of discussion with specialists, leaving her pondering if treatment is worth it after all:

*‘I mean my main interest in the therapy is whether or not it’s worth the risk of increased side effects, so I don’t think there is enough information from many healthcare professionals on either the different types you can take or that risk benefit analysis of… really working out whether or not you should take it in the first place’* (Rose).

Similarly, for Sue, support from specialists should be provided in the form of understanding the survival benefit so women can effectively make an informed decision about HT:

*‘I think treatment should be a little more individualised. But I can understand, you know, I worked in hospitals all my life so I can understand from the other point of view, you’ve got the guidelines, the guidelines is there and you know therefore you’ve to encourage women to follow the guidelines, so it’s a tricky one, is quite tricky, but the women need to know how much the increase survival is to decide if it’s worth it’* (Sue).

#### 3.3.3. Role of the Pharmacist in Managing Brand Changes

Most participants pointed to a consistent way of managing changes in medication brands after considering which healthcare professional would be more suitable for the task. Many women considered pharmacists as their preferred choice:

*‘The pharmacy is probably a good place actually cause they do understand what you are talking about‘* (Diane).

*‘So yeah, pharmacist is probably your best person, is medication related, it is what pharmacists are supposed to be doing, discussing it’* (Helen).

Diane and Helen agree on identifying pharmacists as the best professional to manage their concerns with medications; however, their decisions are informed by different approaches. For Diane is an experiential response based on finding an empathetic pharmacist, while for Helen, who is a pharmacist herself and has inside knowledge on how the NHS works, gives rather a professional type of response. She continues:

*‘I personally wouldn’t recommend having a specific brand prescribed cause it makes life really difficult for everybody. But I would say that anyone who is having difficulties should be advised to keep a diary and see if there is anything that they can pinpoint and it may not be the brand […] but it might be that month it was particularly hot, like it is now, or it might have been you were particularly stressed -work, kids, whatever. […] And if you can find which brand you don’t get on with, that still leaves you quite a few to have rather than just saying I can only have this one, which limits what you can have’* (Helen).

Helen seems to be outlining a very pragmatic way for women to manage the constant changes in brands. Keeping a diary is something that she did herself when not tolerating a particular brand. Her advice is grounded in her dual identity as a pharmacist and as a breast cancer patient, hence her claim: ‘it is difficult for everybody’ i.e., the patient and the pharmacist.

Other participants also concurred that pharmacists are the right professional to manage their medication due to other reasons.

*‘I think the pharmacist because I don’t always want to bother the doctor, the GP with things like this or sometimes you know you can ring up and not get an appointment for two weeks, whereas if you could go to the pharmacist and say, ‘can you make sure when tamoxifen comes in, I get this brand?’ And it can be managed at that level. I think it would make it a lot easier’* (Liz).

This extract captures the easiness with which some patients experienced the changing of medication brands, which contrasts with that narrated by others for whom finding an empathetic pharmacist is ‘trial and error’ or those who considered pharmacists as ‘gate keepers’ of drugs. In the case of Julie, who was not successful in the request for her desired brand, her expectation is for a better interaction and coherence between prescriber and dispenser:

*‘I guess, I guess what would make it easier is if there was more of a connect between the pharmacy and the GP and the oncologist, which there doesn’t really seem to be’* (Julie).

## 4. Discussion

The analysis of the participants’ narratives provides an in depth, ideographic exploration of women with breast cancer who experienced medication brand substitution for HT. This under-researched issue in the medication-taking and adherence literature has been largely discussed in women’s online breast cancer forums whence we identified it in the first place and recruited participants for this study. The wide use of these forums by cancer survivors as a source of support, information and experiential advice makes it a significant space to explore patients’ perceptions and practices given its potential for encouraging behavioral responses, which can be further explored with other methodologies, including IPA, to gain a more comprehensive picture of this phenomenon. The results from this study indicate that many women noticed an impact of brand changes on their physical or emotional wellbeing and that the impact of these brand changes were not always acknowledged by their healthcare teams, leading some to go to significant lengths to advocate for themselves.

Women’s narratives located their experience of medication changes within the broader context of their personal need to undergo HT. The necessity–concerns framework [5] explains that patients weigh up the belief in the need for treatment (necessity) against their concerns about potential negative consequences (side effects and reduced quality of life). Women’s initial decision to undergo HT was later challenged by the management of side effects once they felt they had intensified after changes in medication brands.

At first, participants described the loss of control over treatment because of unawareness of generic switching practices, despite this being common in the UK health system. Later they realized that access to their preferred generic brand was not always possible, and in the process, they often encountered disbelief from healthcare professionals and pharmacists as they undermined women’s claims of new side effects caused by a change in brands. This resonates with other HT studies wherein lack of information about treatment and side effects has been consistently identified as a factor in the qualitative literature of non-adherence [34,35,36,37]. As discussed, lack of information has been reported in studies about generic substitution for other diseases [12,13,17]. Having timely information about the existence of different brands constitutes meaningful knowledge for women to have and a way of offering choice within HT treatment. While evidence suggest that oncologists often made use of ‘implicit persuasion’ during consultations about treatment options, by underreporting and downplaying the impact of HT in patients’ quality of life [38,39], we cannot discern if this approach also supports the view that ‘all generic brands are the same’ or if it is based on education and training. In any case, discussions of HT medications should be balanced with a patient-centered approach, as recommended in NICE guidelines on medicine optimization: which requests that ‘prescribers take into account people’s preferences and values about treatment options that includes the use of medicines’ and that patients have ‘the opportunity to be involved in making decisions about their medicines’ [40].

For some women, changing medication brands and having to manage new side effects challenges their self-efficacy (the belief or confidence in performing an action to achieve a desired outcome). Self-efficacy is an important determinant of medication adherence in regard to two distinct behavioral components: self-efficacy in medication-taking behavior [41,42] and self-efficacy in managing side effects [43]. Both components are modifiable factors and have led to a range of interventions, psychological (cognitive behavioral therapy and behavior change techniques), lifestyle (diet, yoga and physical activities) and pharmacological (antidepressants and the intake of supplements) to control side effects. Our findings highlight that circumstances that affect both components of self-efficacy are sometimes ‘external’ to the individual’s trait and therefore would require novel, health system-level interventions to address it. Crucially, NICE guidelines emphasize that adherence is not the sole responsibility of the individual but rather a deficiency in the health system [44]. Interventions addressing the latter, however, have been scarce, and medication reviews programs, such as the new structured medication reviews [45], have not included cancer patients within the service.

Another finding of this study is that oncologists, pharmacists and GPs have different opinions regarding the benefits of patients sticking to a brand or changing brands to suit patients’ preferences. This results in different practices based on individual, case-by-case responses to changing prescriptions or finding a pharmacist that is ‘willing’ to support patients. As a consequence, women’s experiences seem to differ, as some felt disadvantaged in their care, while others might decide to discontinue treatment if no support is provided for their medication needs. Future studies should explore this further, with a view to improving the quality and consistency of information and support from healthcare professionals. Supporting women in finding a brand suitable for them should help to improve their quality of life but also help women to continue treatment for longer.

### Strengths and Limitations

This is the first study to report on the experiences and perceptions of women with breast cancer whose HT medication brand changed during treatment. We recognize due caution with sample generalizability as is the norm with IPA studies. As explained earlier, IPA samples are homogeneous, and our sample was entirely composed of white British women. Hence, further qualitative studies could explore the experiences of minority ethnic groups, as well as that of women with other protected characteristics, as they might differ. Furthermore, most participants were professional women, with jobs or volunteer experience in the health sector, and results might elucidate variation from women with low levels of education, health literacy and self-efficacy to raise questions about treatment decisions from their cancer team.

## 5. Conclusions

This study has presented in-depth, ideographic findings of the experience of HT medication brand changes (generic substitution) in women with breast cancer. Women reported negative physical and emotional experiences of generic substitutions that is often compounded by healthcare professionals’ lack of information and reassurances, disbelief in the worsening of side effects and inconsistent advice regarding generics, with implications for quality survivorship care. Some women went to great lengths to advocate for themselves and directly source their preferred brand. The inability to stick to a consistent or preferred generic/s can impact women’s ability to manage side effects and ultimately affect their adherence and persistence with treatment. Our findings also expose a perceived lack of shared decision-making and patient-centered approaches regarding HT medication prescribing, which is a substantive concern. Further research is required to develop and test interventions to support medication-taking behavior in women reporting negative experiences after changing medication brands. Tapping into the expertise of pharmacists, as suggested by patients on this study, can inform interventions to improve HT medication consultations in a person-centered manner.

Interventions for pharmacists or other healthcare professionals to support women will be of great benefit to breast cancer survivors. Ensuring that patients can voice their concerns and express their medication preferences is in line with shared decision-making principles. Breast cancer survivors prescribed HT drugs should be involved in the planning and implementation of interventions to improve medication consultations. These interventions should enhance the medication-taking experiences for women prescribed HT.

## Figures and Tables

**Table 1:** Participant characteristics.

Participant (Pseudonym)	Age	Ethnicity	Cancer Stage	Time on HT	Years Prescribed HT	Current HT Status
Anna	52	White British	3	1 yr., 3 mo.	5—maybe 10	continues
Helen	50	White British	3	5 yrs.	5—maybe 10	undecided
Rose	53	White British	?	6 mo.	5	continues
Liz	35	White British	1	3 yrs.	10	continues
Julie	58	White British	2	3 yrs.	10	continues
Mary	71	White British	?	5 yrs.	5	completed
Sue	67	White British	2	1 yr., 3 mo.	5	discontinued
Diane	71	White British	2	2 yrs.	5	continues
Kate	56	White British	2	3 yrs., 5 mo.	indefinitely	continues

HT = hormone therapy; yrs. = years; mo. = months.

**Table 2:** Superordinate and subordinate themes.

Superordinate Themes	Subordinate Themes
Experiencing brand changes	Feeling the change—impact on wellbeing Losing and gaining control over treatment A motivation to continue with treatment
Responsiveness of health care providers	Feeling abandoned by the health system—context of lack of continuity of care Encountering disbelief Feeling lucky
Future expectations	Needing information about brands Seeking specialists’ reassurances Role of the pharmacists in managing brand changes

## Data Availability

The data presented in this study are available on request from the corresponding author. The data are not publicly available due to a risk of compromising confidentiality and participant privacy.

## Supplementary Materials

The following supporting information can be downloaded at: https://www.mdpi.com/article/10.3390/healthcare10122558/s1, Table S1: COREQ checklist.

## References

[1] Cancer Research UK (2021). Breast Cancer Statistics. https://www.cancerresearchuk.org/health-professional/cancer-statistics/statistics-by-cancer-type/breast-cancer#heading-Zero.

[2] Huiart L., Bouhnik A.D., Rey D., Rousseau F., Retornaz F., Meresse M., Bendiane M.K., Viens P., Giorgi R. (2013). Complementary or alternative medicine as possible determinant of decreased persistence to aromatase inhibitor therapy among older women with non-metastatic breast cancer. PLoS ONE.

[3] Hershman D.L., Kushi L.H., Shao T., Buono D., Kershenbaum A., Tsai W.Y., Fehrenbacher L., Gomez S.L., Miles S., Neugut A.I. (2010). Early discontinuation and nonadherence to adjuvant hormonal therapy in a cohort of 8769 early-stage breast cancer patients. J. Clin. Oncol..

[4] Partridge A.H., Wang P.S., Winer E.P., Avorn J. (2003). Nonadherence to adjuvant tamoxifen therapy in women with primary breast cancer. J. Clin. Oncol..

[5] Horne R., Weinman J. (1999). Patients’ beliefs about prescribed medicines and their role in adherence to treatment in chronic physical illness. J. Psychosom. Res..

[6] Moon Z., Moss-Morris R., Hunter M.S., Carlisle S., Hughes L.D. (2017). Barriers and facilitators of adjuvant hormone therapy adherence and persistence in women with breast cancer: A systematic review. Patient Prefer. Adherence.

[7] Toivonen K.I., Williamson T.M., Carlson L.E., Walker L.M., Campbell T.S. (2020). Potentially Modifiable Factors Associated with Adherence to Adjuvant Endocrine Therapy among Breast Cancer Survivors: A Systematic Review. Cancers.

[8] Eraso Y., Stefler D., Moon Z., Rossi L., Assefa S. (2021). Extending Adjuvant Endocrine Therapy for 10 Years: A Mixed-Methods Analysis of Women’s Decision Making in an Online Breast Cancer Forum. Healthcare.

[9] National Institute for Health and Care Excellence (NICE) (2022). British National Formulary. Guidance on Prescribing. https://bnf.nice.org.uk/medicines-guidance/guidance-on-prescribing/.

[10] NICE (2022). British National Formulary. https://www.nice.org.uk/.

[11] Kesselheim A.S., Misono A.S., Shrank W.H., Greene J.A., Doherty M., Avorn J., Choudhry N.K. (2013). Variations in pill appearance of antiepileptic drugs and the risk of nonadherence. JAMA Intern. Med..

[12] Dunne S.S. (2016). What Do Users of Generic Medicines Think of Them? A Systematic Review of Consumers’ and Patients’ Perceptions of, and Experiences with, Generic Medicines. Patient.

[13] Straka R.J., Keohane D.J., Liu L.Z. (2017). Potential Clinical and Economic Impact of Switching Branded Medications to Generics. Am. J. Ther..

[14] MacKrill K., Petrie K.J. (2018). What is associated with increased side effects and lower perceived efficacy following switching to a generic medicine? A New Zealand cross-sectional patient survey. BMJ Open.

[15] Rathe J., Andersen M., Jarbøl D.E., Christensen R.D., Hallas J., Søndergaard J. (2015). Generic Switching and Non-Persistence among Medicine Users: A Combined Population-Based Questionnaire and Register Study. PLoS ONE.

[16] Ström O., Landfeldt E. (2012). The association between automatic generic substitution and treatment persistence with oral bisphosphonates. Osteoporos. Int..

[17] Shrank W.H., Cadarette S.M., Cox E., Fischer M.A., Mehta J., Brookhart A.M., Avorn J., Choudhry N.K. (2009). Is there a relationship between patient beliefs or communication about generic drugs and medication utilization?. Med. Care.

[18] Ma S., Shepard D.S., Ritter G.A., Martell R.E., Thomas C.P. (2020). The impact of the introduction of generic aromatase inhibitors on adherence to hormonal therapy over the full course of 5-year treatment for breast cancer. Cancer.

[19] Winn A.N., Fergestrom N.M., Neuner J.M. (2019). Using Group-based Trajectory Models and Propensity Score Weighting to Detect Heterogeneous Treatment Effects: The Case Study of Generic Hormonal Therapy for Women With Breast Cancer. Med. Care.

[20] Neuner J.M., Kamaraju S., Charlson J.A., Wozniak E.M., Smith E.C., Biggers A., Smallwood A.J., Laud P.W., Pezzin L.E. (2015). The introduction of generic aromatase inhibitors and treatment adherence among Medicare D enrollees. JNCI J. Natl. Cancer Inst..

[21] Hershman D.L., Tsui J., Meyer J., Glied S., Hillyer G.C., Wright J.D., Neugut A.I. (2014). The change from brand-name to generic aromatase inhibitors and hormone therapy adherence for early-stage breast cancer. JNCI J. Natl. Cancer Inst..

[22] Qin X., Huckfeldt P., Abraham J., Yee D., Virnig B.A. (2021). Generic entry of aromatase inhibitors and pharmaceutical access: Initiation of hormonal therapy, timeliness of initiation, and drug choice. Res. Soc. Adm. Pharm..

[23] Zeidan B., Anderson K., Peiris L., Rainsbury D., Laws S. (2016). The impact of tamoxifen brand switch on side effects and patient compliance in hormone receptor positive breast cancer patients. Breast.

[24] Blencowe N.S., Reichl C., Gahir J., Paterson I. (2010). The use of Nolvadex in the treatment of generic Tamoxifen-associated small joint arthralgia. Breast.

[25] Smith J., Flowers P., Larkin M. (2009). Interpretative Phenomenological Analysis: Theory, Method and Research.

[26] Yardley L., Morrison L., Bradbury K., Muller I. (2015). The person-based approach to intervention development: Application to digital health-related behavior change interventions. J. Med. Internet Res..

[27] Tong A., Sainsbury P., Craig J. (2007). Consolidated criteria for reporting qualitative research (COREQ): A 32-item checklist for interviews and focus groups. Int. J. Qual. Health Care.

[28] Breast Cancer Now (2021). Breast Cancer Now Forum. https://forum.breastcancernow.org/.

[29] Muggleton J., Guy H., Howard R. (2015). Breaking the taboo: An interpretative phenomenological analysis of healthcare professionals’ experience of caring for palliative patients with disgusting symptoms. BMJ Support. Palliat. Care.

[30] Spiers J., Smith J.A., Poliquin E., Anderson J., Horne R. (2016). The experience of antiretroviral treatment for Black West African women who are HIV positive and living in London: An interpretative phenomenological analysis. AIDS Behav..

[31] Piekarz H., Langran C., Donyai P. (2021). A phenomenological analysis of the experience of taking medication to prevent a further heart attack. Sci. Rep..

[32] Nizza I.E., Farr J., Smith J.A. (2021). Achieving excellence in interpretative phenomenological analysis (IPA): Four markers of high quality. Qual. Res. Psychol..

[33] Montague J., Phillips E., Holland F., Archer S. (2020). Expanding Hermeneutic Horizons: Working as Multiple Researchers and with Multiple Participants. Res. Methods Med. Health Sci..

[34] Brett J., Boulton M., Fenlon D., Hulbert-Williams N.J., Walter F.M., Donnelly P., Lavery B.A., Morgan A., Morris C., Watson E.K. (2018). Adjuvant endocrine therapy after breast cancer: A qualitative study of factors associated with adherence. Patient Prefer. Adherence.

[35] Moon Z.E., Moss-Morris R., Hunter M.S., Hughes L.D. (2017). Understanding tamoxifen adherence in women with breast cancer: A qualitative study. Br. J. Health Psychol..

[36] Clancy C., Lynch J., Oconnor P., Dowling M. (2020). Breast cancer patients’ experiences of adherence and persistence to oral endocrine therapy: A qualitative evidence synthesis. Eur. J. Oncol. Nurs..

[37] Peddie N., Agnew S., Crawford M., Dixon D., MacPherson I., Fleming L. (2021). The impact of medication side effects on adherence and persistence to hormone therapy in breast cancer survivors: A qualitative systematic review and thematic synthesis. Breast.

[38] Engelhardt E.G., Pieterse A.H., van der Hout A., de Haes H.J., Kroep J.R., van Ufford-Mannesse P.Q., Portielje J.E., Smets E.M., Stiggelbout A.M. (2016). Use of implicit persuasion in decision making about adjuvant cancer treatment: A potential barrier to shared decision making. Eur. J. Cancer.

[39] Eraso Y. (2019). Oncologists’ perspectives on adherence/non-adherence to adjuvant endocrine therapy and management strategies in women with breast cancer. Patient Prefer. Adherence.

[40] NICE (2016). Medicines Optimisation: Quality Standard. www.nice.org.uk/guidance/qs120.

[41] Risser J., Jacobson T.A., Kripalani S. (2007). Development and psychometric evaluation of the Self-efficacy for Appropriate Medication Use Scale (SEAMS) in low-literacy patients with chronic disease. J. Nurs. Meas..

[42] Kimmick G., Edmond S.N., Bosworth H.B., Peppercorn J., Marcom P.K., Blackwell K., Keefe F.J., Shelby R.A. (2015). Medication taking behaviors among breast cancer patients on adjuvant endocrine therapy. Breast.

[43] Shelby R.A., Edmond S.N., Wren A.A., Keefe F.J., Peppercorn J.M., Marcom P.K., Blackwell K.L., Kimmick G.G. (2014). Self-efficacy for coping with symptoms moderates the relationship between physical symptoms and well-being in breast cancer survivors taking adjuvant endocrine therapy. Support. Care Cancer.

[44] NICE (2009). Medicines Adherence: Involving Patients in Decisions about Prescribed Medicines and Supporting Adherence. https://www.nice.org.uk/Guidance/CG76.

[45] NHS (2020). Structured Medication Reviews and Medicines Optimisation: Guidance. https://www.england.nhs.uk/publication/structured-medication-reviews-and-medicines-optimisation/.

